# Phosphatase and Tensin Homolog Immunohistochemical Expression and Promoter Methylation Status in Endometrioid Endometrial Carcinoma and Its Precursor Lesions

**DOI:** 10.7759/cureus.30778

**Published:** 2022-10-27

**Authors:** Sunita Yadav, Annu Makker, Preeti Agarwal, Uma Singh, Seema Nayak, Madhu Mati Goel

**Affiliations:** 1 Pathology, King George's Medical University, Lucknow, IND; 2 Biochemistry, Maharshi Vishwamitra Autonomous State Medical College, Ghazipur, IND; 3 Obstetrics and Gynaecology, King George's Medical University, Lucknow, IND; 4 Laboratory Medicine, Medanta Hospital Lucknow India, Lucknow, IND

**Keywords:** endometrial hyperplasia, promoter methylation, endometroid endometrial carcinoma, pten, immunohistochemistry (ihc)

## Abstract

Background: Endometrioid endometrial carcinoma (EEC) is the most common invasive malignancy of the female genital tract. Despite advances in diagnosis and treatment, the incidence of EEC and mortality related to it have not decreased. Therefore, research is needed to explore the underlying molecular mechanisms of EEC and its precursors to reduce the mortality and societal burden associated with them. Phosphatase and tensin homolog (PTEN) is a tumor suppressor gene most commonly altered in endometrial carcinoma and its precursor lesions. Promoter methylation is a common mechanism for the inactivation of the PTEN tumor suppressor gene.

Methods: This was a prospective nested case-control study involving women aged 35 to 70 years old whose endometrial biopsy and resected samples were obtained for histological diagnosis. Before enrolling a person in the study, signed informed consent was obtained from each individual. The ethics committee for the institute gave its approval to the study protocol. Immunohistochemistry (IHC) was used to measure PTEN expression was measured, and methylation-specific PCR (MSP) was used to determine PTEN promoter methylation status (Bisulfite conversion).

Results: A total of 95 samples were assessed histopathologically, along withPTEN expression and PTEN promoter methylation status. PTEN immunoreactivity was observed in 79% (15/19) of normal proliferative endometrium, and loss of PTEN expression was observed in 73% (27/37) of endometrial hyperplasia with or without atypia and 90% (35/39) of EEC. Methylation analysis showed that the PTEN promoter was completely unmethylated in all normal proliferative endometria and endometrial hyperplasia without atypia. In contrast, the promoter region was methylated in 50% of endometrial hyperplasia with atypia cases and 38.5% of EEC cases.

Conclusion: The loss ofPTEN expression was significantly associated with EEC and precancerous lesions of the endometrium compared to normal proliferative endometria. Methylation analysis also revealed that the frequency of methylation is significant in EEC and endometrial hyperplasia with atypia. Integration of PTEN protein expression along with promoter methylation status elucidates the underlying carcinogenic mechanism. This may help with personalized therapy for EECs and triaging cases of potential precancerous lesions.

## Introduction

Globally, endometrial carcinoma (EC) is the most common invasive cancer of the uterine corpus. It contributes to about 7% of all invasive cancers in women [1]. Though Asian women have a lower incidence of EC than other parts of the world, recent studies have shown an increasing trend in these countries, including India [2,3]. In India, there were 13,328 estimated new cases of EC in 2018, and there were 5010 fatalities. Clinicopathologically, EC is divided into two categories: types I and II. Estrogen dependence characterizes type I carcinomas, which comprise 80% to 85% of all cases.

Most type I carcinomas are well differentiated and mimic proliferative endometrial glands and are referred to as endometrioid endometrial carcinomas (EEC). Type II carcinomas are poorly differentiated tumors and account for approximately 15% of cases of EC. These are usually estrogen-independent [4].

EEC typically arises in the setting of endometrial hyperplasia. Clinicopathological and epidemiological studies have shown the malignant potential of endometrial hyperplasia. Similarly, molecular studies also showed their malignant potential. Endometrial hyperplasia is associated with prolonged estrogenic stimulation of the endometrium, which can be due to endogenous or exogenous hyperestrogenism. So, they are associated with obesity, diabetes mellitus, hypertension, infertility, nulliparity, anovulation, and unopposed estrogen stimulation [5]. The most significant of these is obesity, which is a rapidly increasing problem in India [6]. Endometrial hyperplasia precedes EEC development, as proved by many studies, as they share common predisposing risk factors [7,8]. Carcinogenesis results from the stepwise acquisition of genetic alterations in tumor suppressor genes and oncogenes. Phosphatase and tensin homolog (PTEN) is a tumor suppressor gene that negatively regulates the PI3K-AKT signaling pathway. The most frequently altered gene in estrogen-dependent endometrial carcinoma is PTEN [9,10]. PTEN tumor suppressor gene inactivation is common in endometrial hyperplasia and endometrial carcinoma. According to the study by Ma and Gao [11], methylation of DNA was the most common and important epigenetic modification associated with the development, progression, and metastasis of endometrial carcinoma. Since EEC is the most common invasive malignancy of the female genital tract and there is an increasing trend in the incidence rate, early and accurate detection and prognostication will help to decrease mortality and morbidity. The common treatment modalities for EEC are hysterectomy and postoperative radiotherapy. However, there is still a lack of effective treatment for recurrences and progression. Therefore, integrated histological diagnosis with immunohistochemistry (IHC) and molecular analysis may help predict recurrences and progression, thereby reducing mortality and the societal burden of the EEC.

## Materials and methods

Study design

It was a prospective nested case-control study. The study was carried out at the department of pathology of a tertiary-care teaching hospital in northern India. The study was conducted between February 2017 and March 2018. The endometrial biopsies included surgically resected specimens, and clinical parameters were recorded. Based on histopathological reports, biopsies were classified as normal endometrium, hyperplastic endometrium, and endometrioid endometrial carcinoma [12]. Prior to the enrolment in the study, written informed consent was obtained from all the study participants. Patients diagnosed with other malignancies outside the uterus and all endometrial malignancies other than EEC were excluded. Each sample was divided into two parts. The first part was collected in 10% neutral formalin for routine histopathology and immunolocalization of PTEN. The second portion was collected in PBS (pH = 7.4) for the study of promoter methylation of the PTEN tumor suppressor gene. The study protocol was approved by the ethical committee of the Medical University (Ref. no. 78th ECM IIBMD-Ph.D./P2).

Laboratory techniques and procedures

Histopathological Diagnosis

The tissues were obtained at 10% formalin and processed as per standard paraffin embedding techniques. Sections were stained with hematoxylin and eosin (H&E) for the histological examination of the tissue [12].

Immunohistochemistry of PTEN

PTEN IHC staining was done on selected sections of endometrial lesions that were normal, hyperplastic, and cancerous. Sections were treated with murine monoclonal anti-PTEN antibody clone 6H2.1(Millipore, 04-035) at a 1:100 dilution after microwave antigen retrieval, and they were then incubated for an overnight period at 4 °C. The sections were then rinsed, exposed to Novo Link (Leica Biosystems Newcastle Ltd, UK) Polymer for an hour at room temperature, and then treated with 3,3'-diaminobenzidine tetrahydrochloride (DAB). PTEN-stained cells were counted, and their intensity was measured using semi-quantitative scoring [13]. Immunointensity was rated on a scale of 0 (no staining), 1 (light staining), 2 (low intermediate), 3 (high intermediate), and 4 (darkest brown stain). The percentage of staining was graded on a scale of 1: 0-25% of the cells stain positively, 2: 26-50% of the cells stain positively, 3: 51-75% of the cells stain positively, and 4: 76-100% of the cells stain positively. Results ranged from 0 to 16 when the immunointensity and immunopositivity values were multiplied. Samples were classified as negative in the final evaluation for sections scoring 0-3 and positive for sections scoring ≥4.

DNA Isolation and Methylation-Specific PCR

DNA isolation: The Pure Link Genomic DNA kit (catalog no. K1820, Invitrogen, Life Technologies, CA, USA) was used to extract and purify genomic DNA in accordance with the manufacturer's recommendations. The integrity of the extracted DNA was examined through electrophoresis on a 0.8% agarose gel containing 0.5 g/ml of ethidium bromide. The high molecular weight gDNA appeared as a single band near the well when viewed under ultraviolet light (254 nm wavelength). Isolated DNA was quantified using Qubit dsDNA broad-range assay kits (Invitrogen, Life Technologies, CA; catalog no. Q32853) on a Qubit 2.0 fluorometer.

Bisulfite treatment and methylation-specific PCR: The bisulfite conversion of gDNA (200-500 ng) was performed using a commercially available EZ DNA Methylation-GoldTM Kit (Cat no. #D5005, Zymo Research, Irvine, CA, USA). Promoter methylation was analyzed by methylation-specific PCR (MSP), as described by Missaoui et al. [14]. Real-time based MSP was performed using SYBR premix ExTaq (Applied Biosystems, Waltham, MA, USA), primers specific for the methylated (M) or unmethylated (U) sequences, and bisulfite-modified DNA on an Applied Biosystems Step One Real-time PCR Detection System (ABI, Foster, CA) with optimized thermal conditions, followed by a final step of melt curve (One cycle of 95 °C - 15 secs, 60 °C - 60 secs, 95 °C - 15 secs with a ramp rate of 0.4). Methyl Primer Express v1.0 Software (Applied Biosystems) and meth primer [15], a free online primer design tool, were used for designing methylated and unmethylated primers. Specific primer sequences (methylated and unmethylated) were used for PTEN (Table 1). A sample was regarded as methylation-negative if the PCR product was obtained only with the U set and methylation-positive if the PCR product was obtained with the M set or with both the U set and the M set. Human HCT116 DKO non-methylated DNA and methylated DNA were used as negative and positive controls, respectively (Cat no. #D5014, Zymo Research, Irvine, CA, USA). MSP was performed in duplicate on all specimens. PCR products were run on 2% agarose gels and visualized after ethidium bromide staining.

**Table 1:** Details of primers used for methylation specific PCR M: methylated, U: unmethylated, F: forward primer, R: reverse primer

Gene (Accession No.)		Primer sequence		Amplicon (bp)
PTEN (Genbank AF143312)	M	F	GGCGGCGGTCGCGGTTC	71
		R	GACTCCCCGAAAACGCTAC	
	U	F	GAGAGATGGTGGTGGTTGT	78
		R	AACTCCCCAAAAACACTACC	

Statistical analysis

Data were first entered into the pretested structured proforma and then entered into an MS Excel sheet (Microsoft® Corp., Redmond, WA). The final analysis was done by SPSS software version 24. The categorical variables were compared with Pearson’s chi-square tests, and a p-value <0.05 was considered statistically significant.

## Results

**Figure 1:**
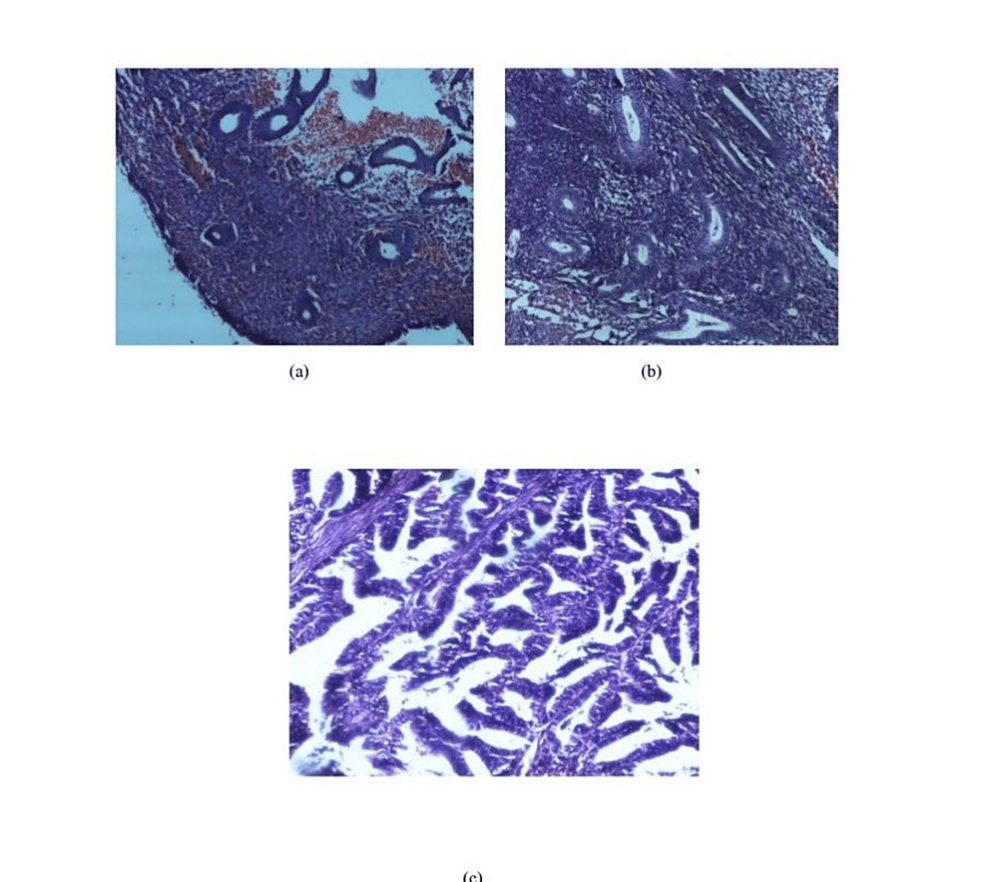
Hematoxylin and eosin stained sections' photomicrograph Photomicrographs of Hematoxylin and Eosin stained sections of (a) normal proliferative endometrium, (b) precancerous endometrium (endometrial intraepithelial neoplasia), and (c) endometrioid endometrial carcinoma

A total of 95 endometrial samples were assessed. Of these, 39 were EEC, 37 were endometrial hyperplasia, and 19 were normal proliferative endometria (Figure 1). The most frequent age group in the present study was 51-60 years for EEC and 41-50 years for precancerous lesions. Most precancer patients (59%) were within 49 years of age, and in the cancer group (82%), the majority were above 49 years of age. Both were statistically significant. The most frequent complaints in our patients were bleeding per vaginum (95%) and pain in the abdomen (32%) in EEC cases, while abnormal uterine bleeding (43%), menorrhagia (14%), excessive bleeding during menses (14%), and pain in the abdomen (10%) in precancer cases. Figure 1 shows the immunohistochemical analysis of PTEN expression.

**Figure 2:**
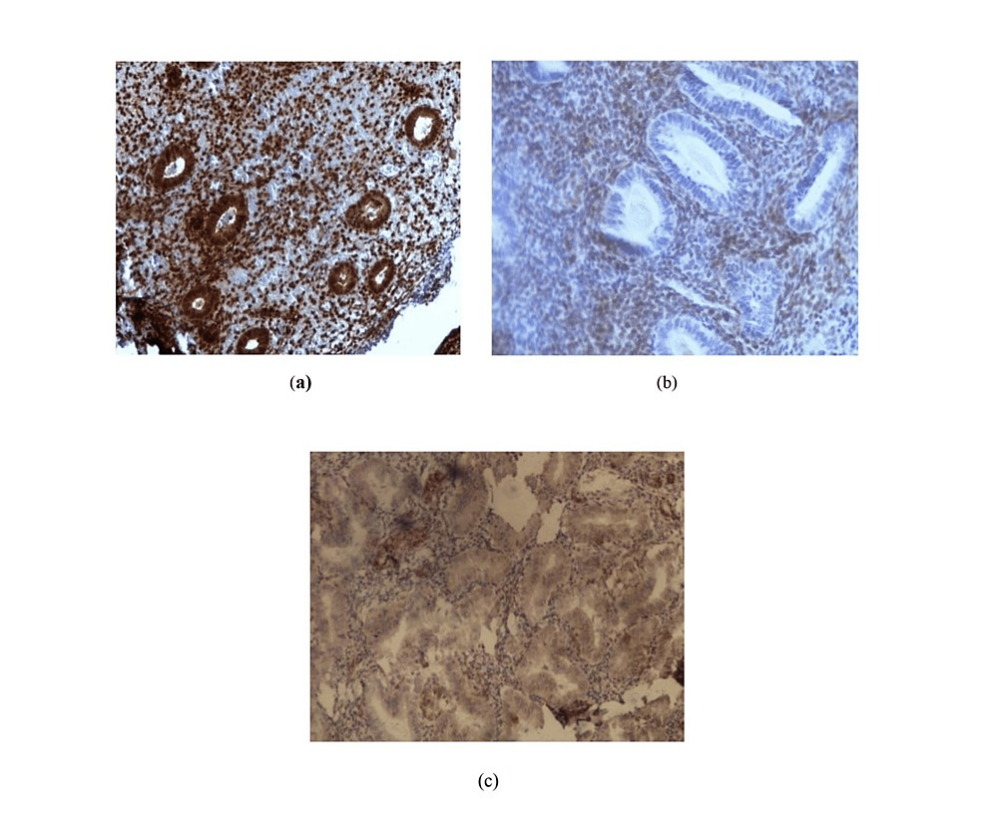
Photomicrograph of immunohistochemical expression of PTEN Immunohistochemical localization of PTEN (a) normal proliferative endometrium showing strong nuclear and cytoplasmic staining in glandular epithelium as well as stroma; (b) precancerous endometrium showing loss of nuclear PTEN in gland and stroma; (c) endometrioid endometrial carcinoma showing loss of both nuclear and cytoplasmic PTEN in glands as well as in stroma

Immunohistochemical expression of PTEN is shown in Figure 2.

PTEN immunoreactivity was observed in 79% (15/19) of the glandular epithelium of normal proliferative endometrium. Loss of PTEN expression was observed in 73% (27/37) of endometrial hyperplasia with or without atypia and 90% (35/39) of EEC (Table 2).

**Table 2:** Demography, immunohistochemical expression, and methylation status of PTEN in cancer, precancer, and control *Fisher exact test

S.No.	Variables		Groups				
			Control (N=19) (a)	Precancer (N=37) (b)			Cancer (N=39) (c)
1.	Age (year)	Mean ± SD	39.63±8.03	45.51±8.61			57.13±10.62
		Median	42	46			60
		<49	17 (89%)	22 (59%)			7 (18%)
		>49	2 (11%)	15 (41%)			32 (82%)
		χ^2^		5.349 (a vs b)			26.94 (a vs c)
		p-value		0.0207 (a vs b)			0.00001(a vs c)
2.	PTEN Expression (IHC)		Control (N=19) (a)	Endometrial hyperplasia without atypia (N=27) (b.1)	Endometrial Hyperplasia with atypia (N=10) (b.2)	Total (N=37) (b)	Cancer (N=39) (c)
		Present	15 (79%)	8 (29.5%)	2 (20%)	10 (27%)	4 (10%)
		Absent	4 (21%)	19 (70.5%)	8 (80%)	27 (73%)	35 (90%)
		χ^2^		10.84 (a vs b.1)	9.38 (a vs b.2)	13.69 (a vs b)	27.36 (a vs c)
		p-value		0.0009 (a vs b.1)	0.002 (a vs b.2)	0.000215 (a vs b)	0.0001 (a vs c)
3.	PTEN promoter methylation	Methylation present	0 (0%)	0 (0%)	2 (50%)	2 (20%)	5 (38.5%)
		Methylation Absent	14 (100%)	6 (100%)	2 (50%)	8 (80%)	8 (61.5%)
		Total (N)	14 (100%)	6 (100%)	4 (100%)	10 (100%)	13 (100%)
		p-value*		NA	0.039 (a vs b.2)	0.163 (a vs b)	0.0159 (a vs c)

Within the precancer group, loss of PTEN was observed in 80% of cases of endometrial hyperplasia with atypia and 70.4% of cases of hyperplasia without atypia. Only 21% of cases of normal control demonstrated a focal, heterogenous decrease of PTEN expression.Compared to the normal control group, there was a statistically significant reduction of PTEN expression in the glandular epithelium of the EEC (χ2 27.36, p-value = 0.0001) and precancer (χ2 13.69, p-value = 0.000215) groups.

Analysis of epigenetic silencing of PTEN by promoter methylation study

**Figure 3:**
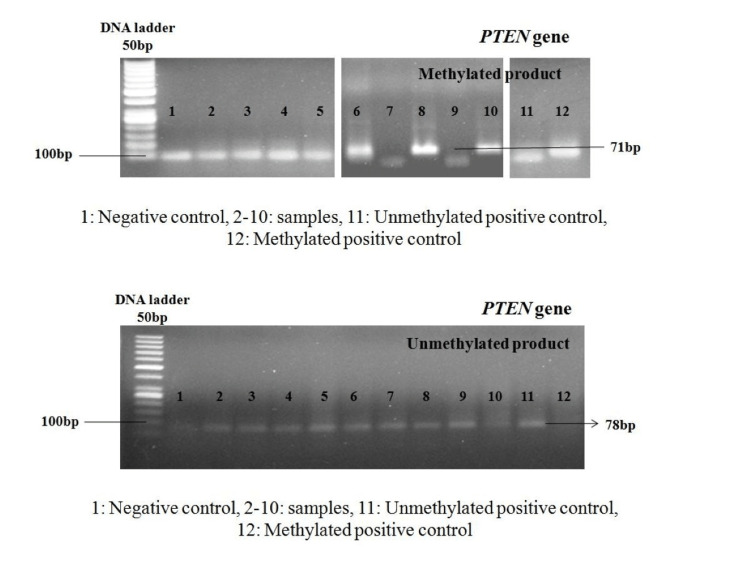
Status of promoter methylation of PTEN: methylated and unmethylated products

Methylation analysis was performed by using methylation-specific PCR for PTEN gene prompter regions in 37 endometrial samples (14 control, 13 cancers, and 10 precancers, comprising six endometrial hyperplasias without atypia and four hyperplasias with atypia). To visualize PCR products, 2% agarose gel electrophoresis was used. The PTEN promoter was completely unmethylated in all controls (14/14, 100%) and all endometrial hyperplasia without atypia (6/6, 100%). However, the promoter region was methylated in 50% (2/4) of endometrial hyperplasia with atypia cases and 38.5% (5/13) of EEC cases. Alteration of promoter methylation of the PTEN tumor suppressor gene was statistically significant in EEC (p = 0.0159) and endometrial hyperplasia with atypia (p = 0.039) as compared to normal control. Gel pictures of methylated and unmethylated products are shown in Figure 3.

## Discussion

In the current study, it was observed that the majority of EEC (35/39, 90%) and endometrial hyperplasia with or without atypia (27/37, 73%) were negative (loss of expression) for PTEN immunoreactivity. The results of IHC analysis of PTEN expression in endometrial hyperplasia have been ambiguous and varied across various published studies [4,16]. In the present study, we found a significantly higher (73%) loss of PTEN expression in hyperplasia compared to normal proliferative endometrium. This could be because endometrial hyperplasia usually precedes EEC and has a similar pattern of molecular alterations to that found in EEC, and PTEN inactivation may be an initial event in the process of carcinogenesis. In cases of hyperplasia without atypia, we found a 70% loss of PTEN immunoreactivity, which may be due to molecular alteration preceding the morphological changes, supported by a study by Mutter et al. [17]. They observed that 40% of the histologically normal-looking perimenopausal endometrium showed loss of PTEN expression. Erkanli et al. [18] reported a less homogenous pattern with a few scattered PTEN null glands interposed among PTEN-positive glands in proliferative endometrium. However, other studies did not observe a loss of PTEN expression in hyperplasia without atypia [18,19]. Aguilar et al. [20] observed aberrant PTEN expression in 50.5% of endometrial hyperplasia cases. Allithy et al. [19] did not observe a loss of PTEN expression in hyperplasia without atypia, while most atypical hyperplasia (27/29, 93%) showed mild to moderate loss of PTEN expression. Loss of PTEN immunoreactivity was found in 25% (2/8) of cases of hyperplasia with atypia by Sarmadi et al. [8]. Shanmugapriya et al. [21] highlighted the immunohistochemical evaluation of PTEN in endometrial hyperplasia and EECs as a useful screening method to detect precancerous lesions for early diagnosis of EECs.

In the present study, the loss of PTEN expression in EEC was 90%. Similar to the study by Mutter et al. [4], which reported that 97% of cases of EEC showed a loss of PTEN expression. However, Sarmadi et al. [8] and Sal et al. [22] reported a loss of PTEN expression slightly less (52% and 61%) in cases of EEC than in the present study. Although Allithy et al. [19] found that none of the EEC showed strong PTEN expression, a finding similar to our study. The variation in the loss of PTEN expression in various studies could be due to various stages of disease progression, different age groups, variability in the sampling, and the use of different types of antibodies (polyclonal or monoclonal). Monoclonal antibodies were associated with more accurate results than polyclonal [23]. PTEN gene alteration is most commonly found in type I EC; therefore, case selection is the most important factor influencing the variation in PTEN expression.

In addition to mutation and loss of heterozygosity, epigenetic alteration is most frequently associated with PTEN inactivation [24]. In the early stages of cancer, DNA hypermethylation of tumor-suppressor genes is a prevalent molecular change, and it can be highlighted as a biomarker in the detection and treatment [25]. In the present study, methylation analysis showed that the PTEN promoter was completely unmethylated in all controls and endometrial hyperplasia without atypia. However, the promoter region was methylated in 50% of the endometrial hyperplasia with atypia cases and 38.5% (5/13) of the EEC cases. In agreement with the present study, Ghazanfari et al. [26] observed 28.57% PTEN promoter methylation in endometrial tissue, and DNA obtained from blood samples of 11.54% of EEC patients showed promoter methylation. Salvesen et al. [9] found that 19% of EC cases showed promoter methylation and also postulated that metastatic disease and microsatellite instability were significantly associated with the promoter methylation status of EC. Inactivation of the PTEN gene in EEC appears to be frequently associated with PTEN methylation, in addition to mutations and loss of heterozygosity. The loss of PTEN can be explained by our methylation results; i.e., increased methylation of the PTEN promoter gene could be the reason for the downregulation of PTEN. Altered PTEN expression in EEC was associated with PTEN promoter methylation.

In the present study, PTEN promoter methylation was observed in 50% of endometrial hyperplasia with atypia, while hyperplasia without atypia was completely unmethylated. Not much evidence is available on PTEN promoter methylation status in endometrial hyperplasia cases. The risk of progression of non-atypical endometrial hyperplasia to carcinoma is <5%, while it is almost 30% for atypical hyperplasia. Sun et al. [27] have suggested that methylation of the promoter region of the PTEN gene is an important genetic derangement in endometrial proliferative conditions, particularly in the development of cytologic atypia in endometrial hyperplasia.

Allison et al. [28] showed that loss of PTEN expression alone or in combination with a particular histological feature had the most consistency to make a clear distinction between normal, endometrial hyperplasia, and EEC. However, loss of PTEN expression alone is unable to predict the progression of endometrial hyperplasia and EEC. Because of that, the underlying molecular mechanism of PTEN inactivation must be explored for effective personalized therapy for EEC and screening of potential precancerous lesions.

In the present study, a significant proportion of endometrial tissues with hyperplasia (73%) and EEC (90%) had lost PTEN expression and also showed promoter methylation. The increased promoter methylation of PTEN could be the reason for the loss of PTEN protein expression. However, loss of PTEN expression was also observed in endometrial hyperplasia without atypia, but none of the cases showed promoter methylation. This might be attributed to a difference in the underlying molecular mechanism in PTEN gene silencing [24]. While in hyperplasia with atypia, 80% of cases showed loss of PTEN immunostaining, and promoter methylation was also observed in 50% of cases. Previous studies have shown that endometrial hyperplasia with atypia has a potential risk of developing into EEC in the future. But on the basis of histopathology and PTEN immunostaining alone, it is difficult to predict accurately. Therefore, the integration of the PTEN promoter methylation and PTEN immunostaining can be used as a better predictor of progression. This combination can also be used as a screening tool in endometrial hyperplasia cases for detecting potentially precancerous lesions. Therefore, understanding the underlying mechanism of the inactivation of PTEN function in EEC offers a window of opportunity for improved personalized therapy. Although this study provides information regarding PTEN promoter methylation and protein expression in EEC and endometrial hyperplasia, more studies with larger sample sizes will be essential for a better understanding of the role of epigenetics in the progression of precancerous endometrial lesions to cancer.

## Conclusions

The present study showed that loss of PTEN expression was observed to be significantly associated with EEC and precancerous lesions as compared to controls. Methylation analysis also revealed that the frequency of methylation was significantly upregulated in EEC and endometrial hyperplasia with atypia. Therefore, the integration of PTEN protein expression along with promoter methylation status clearly elucidates the underlying carcinogenic mechanism. This will offer a window of opportunity for improved personalized therapy by inhibitors of enzymes that control epigenetic modifications. This combination can be used as a screening tool in endometrial hyperplasia cases to detect potentially precancerous lesions.
